# Increased Urothelial Cell Apoptosis and Chronic Inflammation Are Associated with Recurrent Urinary Tract Infection in Women

**DOI:** 10.1371/journal.pone.0063760

**Published:** 2013-05-15

**Authors:** Fei-Chi Chuang, Hann-Chorng Kuo

**Affiliations:** 1 Department of Obstetrics and Gynecology, Chang Gung Memorial Hospital, Kaohsiung Medical Center, Kaohsiung, Taiwan; 2 Department of Obstetrics and Gynecology, Chang Gung University College of Medicine, New Taipei, Taiwan; 3 Department of Urology, Buddhist Tzu Chi General Hospital, Hualien, Taiwan; 4 Department of Urology, Buddhist Tzu Chi University College of Medicine, Hualien, Taiwan; Northwestern University, United States of America

## Abstract

**Objective:**

This study was designed to investigate whether increased urothelial cell apoptosis and chronic inflammation might contribute to recurrent urinary tract infection (UTI) in women.

**Methods:**

The bladder biopsy specimens were collected from thirty women with recurrent UTI and ten controls. The bladder biopsies were performed at one to two months after UTI episode had been completely resolved and urine analysis and urine culture all showed negative. Immunofluorescence staining of the adhesive protein E-cadherin, mast cell and TUNEL were performed in all the bladder specimens. In addition, western blots were also performed to analyze the inflammatory proteins (phospho-p38, tryptase) and apoptotic protein (Bax) in the bladder mucosa specimens between patients with recurrent UTI and controls.

**Results:**

Immunofluorescence staining showed significantly lower E-cadherin in the recurrent UTI bladder tissue compared with the controls (25.4±8.9 v 42.4±16.7, p<0.0001). The mast cell expression was significantly stronger in the recurrent UTI bladder tissue compared with the controls (2.5±1.8 v 1.3±1.2, p = 0.046). TUNEL staining revealed a significantly higher numbers of apoptotic cells in the recurrent UTI bladder tissue compared with the control bladder tissue (1.5±1.8 v 0.08±0.3, p<0.0001). Western blot analysis also showed that the expressions of tryptase and Bax increased in five recurrent UTI specimens compared with two normal control specimens.

**Conclusion:**

Chronic inflammation, urothelial cell apoptosis and impairment of barrier function of urothelial cells might contribute to recurrent UTI in women.

## Introduction

Recurrent urinary tract infection (UTI) is a very bothersome and a popular problem in the urogynecology clinical practice. According to the IUGA/ICS joint report on the terminology for female pelvic floor dysfunction, recurrent UTI is defined as at least three symptomatic and medically diagnosed UTI in the previous 12 months. The previous UTI(s) should have resolved prior to a further UTI being diagnosed. Recurrent UTI is one of the most common diagnoses for female pelvic floor dysfunction [1].

Interstitial cystitis/bladder pain syndrome (IC/BPS) is a known chronic inflammatory disorder of the urinary bladder. Histologic study showed infiltration of mast cells in IC/BPS bladders and suggested that the disease is mediated by an abnormality of the immune system [2], [3]. A wide consensus has been reported that primary urothelial lining defects play an important role in chronic cystitis and bladder oversensitivity [4]. Our study group has shown that abnormal urothelial barrier function is significantly associated with chronic inflammation and possibly the causative factor of increased urothelial apoptosis [5]. Overactive bladder (OAB) is another subject to be linked to chronic bladder inflammation. Some inflammatory biomarkers such as nerve growth factor (NGF), cytokines and serum C-reactive protein are increased in patients with OAB and those with IC/BPS [6]–[12].

Patients with recurrent UTI might also have bladder irritative symptoms. Previous studies have revealed that patients with recurrent UTI have elevated urinary NGF, suggesting chronic inflammation is present in the bladder of these patients after resolution of UTI [6]. Based on these knowledge, we hypothesized that chronic inflammation might reside in the bladder wall, which might also cause urothelial dysfunction and defective barrier function. UTI might be easy to recur in these patients with residual chronic bladder inflammation. This study was designed to investigate whether increased urothelial cell apoptosis and chronic inflammation may contribute to recurrent UTI in women.

## Materials and Methods

The bladder biopsy specimens were collected from thirty women with recurrent UTI and ten controls. Recurrent UTI was defined as at least three symptomatic and medically diagnosed UTI in the previous 12 months. All patients were treated actively according to the latest urine culture and followed by antimicrobial prophylaxis for at least 1 month. The bladder biopsies were performed at one to two months after the UTI episode had been completely resolved and urine analysis and urine culture all showed negative. The patients’ lower urinary tract symptoms at bladder biopsy were also recorded. Patients were divided to subgroups with or without bladder irritative symptoms. The women of control group were the cases of stress urinary incontinence and the specimens were taken during anti-incontinence surgery.

This study was approved by the Institutional Review Board and Ethics Committee of the hospital. Each patient was informed about the study rationale and procedures and written informed consent was obtained before the bladder biopsy procedures.

The bladder biopsies were taken from the mucosal layer and were obtained at the lateral and posterior walls about 2 cm above the ureteral orifices. Totally four pieces of bladder biopsy specimens were taken, one was sent to the pathology department to exclude the possibility of carcinoma in situ, the other three specimens were embedded in optimal cutting temperature (OCT) medium and stored frozen with liquid nitrogen at −80°C for additional investigations. The biopsy procedures and specimens preparing were the same in the control group.

The bladder mucosa of patients with recurrent UTI and that of control patients were investigated for urothelial apoptosis by TUNEL assay, urothelial junction was assessed by protein E-cadherin expression, and mast cell activation by tryptase level. Immunofluorescence staining of the adhesive protein E-cadherin, mast cell and TUNEL were performed in all the bladder specimens. The urinary bladder specimens were immersed and fixed for 1 hour in an ice-cold solution of 4% formaldehyde in phosphate-buffered saline (PBS) (pH 7.4). They were then rinsed with ice-cold phosphate-buffered saline (PBS) containing 15% sucrose for 12 hours. The biopsy specimens were embedded in OCT medium and stored at −80°C. Four sections per specimen were cut using a cryostat at a thickness of 8 µm and collected on new silane III-coated slides (Muto Pure Chemicals Co. Ltd, Tokyo, Japan). The sections were post-fixed in acetone at −20°C and blocked with rabbit serum. The sections were incubated overnight at 4°C with primary antibodies to anti-human E-cadherin (BD Biosciences, San Jose, CA, USA) or anti-human tryptase (Chemicon, Temecula, CA, USA). After rinsing the sections with 0.1% Tween-20 in PBS, anti-rabbit conjugated fluorescein isothicocyanate secondary antibodies (DakoCytomation, Denmark A/S) were applied to the sections and incubated for I hour. Finally, the sections were counterstained with DAPI (Sigma Chemical Co., St. Louis, MO, USA).

Next, the sections were stained using a terminal deoxynucleotidyl transferase (TdT)-mediated deoxyuridine triphosphate nick end labeling staining (TUNEL) assay kit. The sections were incubated with 100 µL 20 µg/ml proteinase K (Calbiochem, Darmstadt, Germany) at room temperature and washed with PBS. The samples were covered with TdT equilibration buffer (Calbiochem) and incubated at room temperature for 30 minutes. After carefully blotting the 1X TdT equilibration buffer from the specimens, we applied TdT labelling reaction mixture (Calbiochem) onto the specimens and incubated them for 90 minutes at 37°C. After washing with Tris-buffered saline, the specimens were mounted using fluorescein-FragEL mounting media (Calbiochem). The total cell population was visualized using a 330–380 nm filter for 4,6-diamidino-2-phenylindole (DAPI), and the fluorescein-labeled nuclei were visualized using a standard fluorescein filter (465–495 nm) of the Axiovert 200 Inverted Microscope (Zeiss, Thornwood, NY).

In addition, western blots were also performed to analyze the inflammatory proteins (phospho-p38 and tryptase) and apoptotic protein (Bax) in bladder mucosa specimens between five patients with recurrent UTI and two controls. The bladder biopsy specimens were homogenized by liquid nitrogen and the primary urothelial cells were washed twice in ice-cold phosphate-buffered saline and then lysed for 10 minutes on ice using PRO-PREP Protein Extraction solution (iNtRON Biotechnology, Gyeonggi-do, Korea) supplemented with protease inhibitor cocktail and phosphatase inhibitor cocktail (Roche Diagnostics, Mannheim, Germany). The proteins were separated on sodium dodecyl sulfate-polyacrylamide gel electrophoresis, and phospho-p38, tryptase, Bax (Cell Signaling Technology, Danvers, MA) were evaluated using Western blotting and singα-tubulin (Cell Signaling Technology) as a loading control.

The results of immunofluorescence (E-cadherin, mast cell, TUNEL) were quantified by counting the positive cells/total cells per area unit (4 µm^2^), and were shown as the percentage of positive cells per 100 total cells. The intensities of proteins E-cadherin and Western blots were quantified using Image J processing [13]. The expressions of E-cadherin, mast cell and urothelial apoptosis were compared between the recurrent UTI bladder tissue and the control bladder tissue, and between patients with irritative bladder symptoms. Statistical analysis was performed using the Kruskal-Wallis test. All calculations were performed using SPSS for Windows, version 10.0. A *P* value <0.05 was considered statistically significant.

## Results

**Figure 1:**
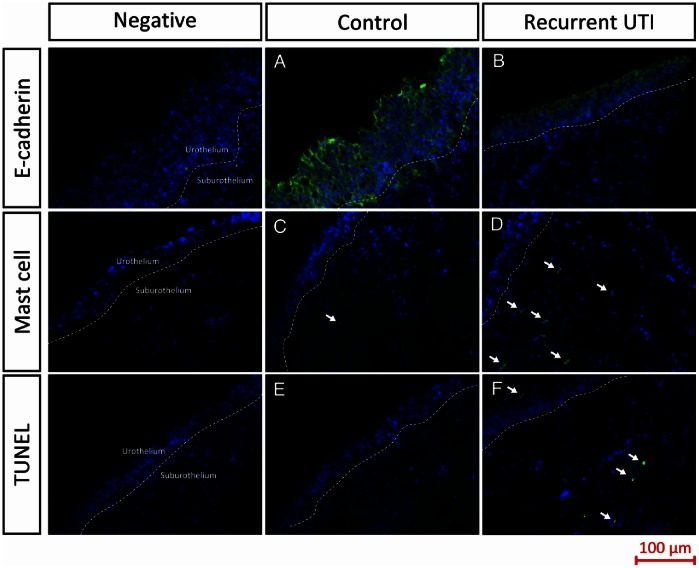
Expressions of E-cadherin, mast cell and TUNEL in the controls and patients with recurrent urinary tract infection (Recurrent UTI). E-cadherin, mast cells and TUNEL staining is green and DAPI staining is blue.

The expression and location of E-cadherin were detected by immunofluorescence, and quantified using Image J processing. E-cadherin immunolabelling was widely distributed in the cell-cell junctions in the superficial layers of the urothelium of the normal control specimen, suburothelial layers showed no immunoreactivity for E-cadherin **(** **Fig. 1** **)**. However, the expression of E-cadherin in the superficial layers of the urothelium of the patients with recurrent UTI was much weaker than the controls **(** **Fig. 1** **)**. **Table 1** showed significantly lower E-cadherin in the recurrent UTI bladder tissue compared with the controls (25.4±8.9 v 42.4±16.7, the fluorescence intensity per 4 µm^2^, p<0.0001).

**Table 1:** Expression of E-cadherin, mast cell and TUNEL in bladder specimens of control and recurrent UTI patients.

	Control(n = 10)	Recurrent UTI (n = 30)	P-value
**E-cadherin**	42.4±16.7	25.4±8.9	<0.0001
**Mast cell**	1.3±1.2	2.5±1.8	0.046
**TUNEL**	0.08±0.3	1.5±1.8	<0.0001

Data are expressed as mean ± SD numbers of positive cells per 100 cells, for E-cadherin data are expressed as mean ± SD pixel of image per area unit (µm^2^).

Mast cell activation was shown by immunofluorescence cytoplasmic staining in the bladder specimen. Some mast cell expression was observed in the suburothelium of both the control and recurrent UTI specimens **(** **Fig. 1** **)**. The quantified results of mast cell expression was significantly stronger in the recurrent UTI bladder tissue compared with the controls (2.5±1.8 v 1.3±1.2, p = 0.046) **(** **Table 1** **)**. TUNEL staining revealed a significantly higher numbers of apoptotic cells in the recurrent UTI bladder tissue compared with the control bladder tissue (1.5±1.8 v 0.08±0.3, p<0.0001). **(** **Table 1** **)** There were almost no apoptotic cells (TUNEL staining positive) in the control tissue, however some apoptotic cells were distributed in the urothelium as well as suburothelium of recurrent UTI bladder tissue **(** **Fig. 1** **)**.

**Figure 2:**
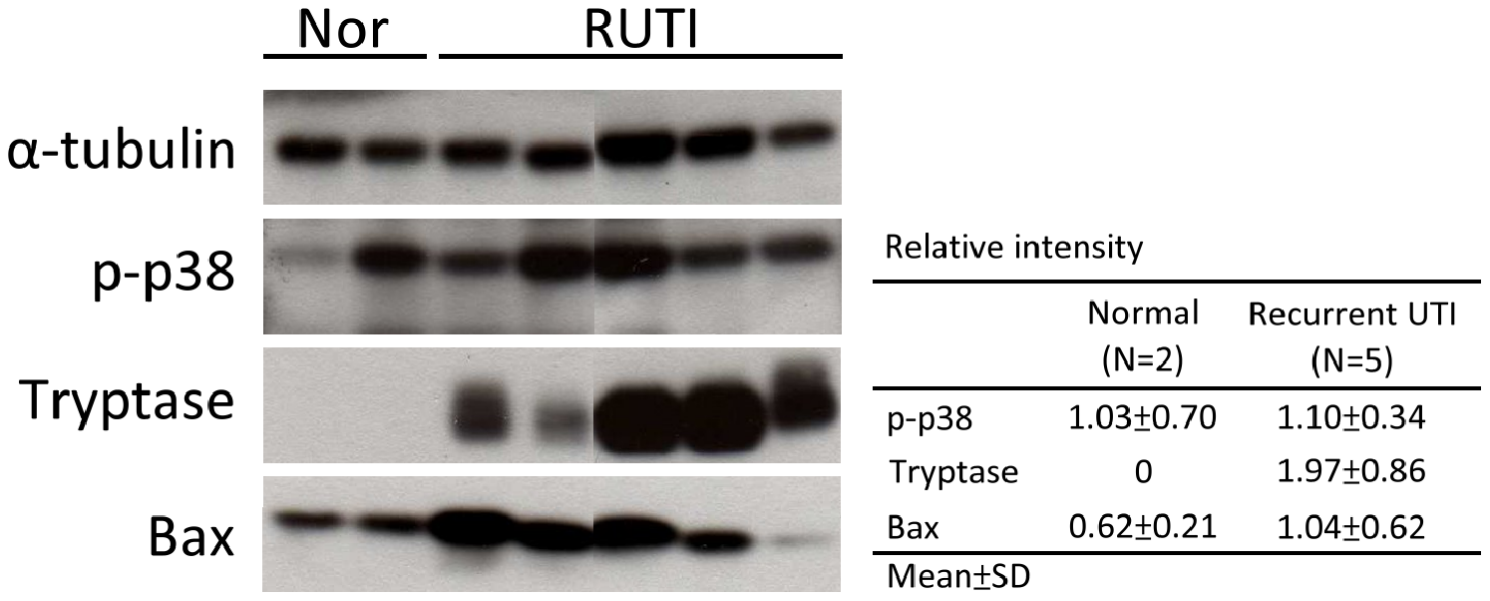
Western blot analysis of expression of phospho-p38, tryptase and Bax in 5 patients with recurrent UTI (RUTI) compared with 2 normal controls (Nor). Relative intensity of phospho-p38, tryptase and Bax in the normal and recurrent UTI were expressed as folds compared with α-tubulin.

The tissue lysates derived from the control (n = 2) and recurrent UTI (n = 5) bladder samples were subjected to Western blot analysis for the measurement of phospho-p38 and trypatse to confirm the inflammatory events and Bax protein expression to prove the apoptotic process. Western blot analysis showed that the expressions of tryptase (about 2.0 folds) and Bax (about 1.67 folds), but not phospo-p38, increased in the recurrent UTI specimens compared with the normal control specimen. **(** **Fig. 2** **)**.

## Discussion

The present study showed that the expressions of the inflammatory proteins and urothelial cell apoptosis were remarkable and the barrier function of urothelium was impaired in recurrent UTI cases. These histopathology might contribute to recurrent UTI in women.

Immunofluorescence staining showed significantly lower E-cadherin in the recurrent UTI bladder tissue compared with the controls, suggesting the barrier dysfunction of urothelium in recurrent UTI patients. The role of adhesive protein E-cadherin in the pathophysiology of IC/BPS has been investigated, it was demonstrated that E-cadherin is associated with bladder sensation and barrier function [5], [14].

In the present study, patients with recurrent UTI had a significantly stronger mast cell expression compared with the normal controls, implied the existence of chronic inflammation in the urothelium. Mast cells, best known for their role in allergic inflammation, are an important source of several inflammatory mediators, including proteases and vasoactive amines such as histamine [15]. Mast cells are considered as crucial effector cells of the immune response implicated in the pathogenesis of IC/BPS [3], [16], [17]. A previous study found increasing mast cell infiltration in both OAB and IC/BPS bladder wall, demonstrating chronic inflammation is involved in pathogenesis of both diseases [17]. Also a closed association between mast cell activity and decreased E-cadherin expression was proved in one IC/BPS study [5].

One recent study demonstrated localized production of mast cell interleukin-10 resulted in suppressed humoral and cell-mediated responses and bacterial persistence. Tissue-resident mast cells not only orchestrate the early innate immunity during bladder infection, they subsequently play a tissue-specific immunosuppressive role which might have association with the recurrent UTI [18]. This observation might explain the mast cell mediated inflammation and related urothelial dysfunction in recurrent bladder infection.

Our study revealed that apoptosis number in the recurrent UTI bladder tissue increased significantly compared with control. Apoptosis is a major form of cell death, characterized initially by a series of stereotypic morphologic changes [19]. It was been demonstrated that the change in the balance between apoptosis and hyperplasia plays a role in the development of diabetic cystopathy [20]. Our previous research indicated that inflammatory and apoptotic events coexisted in the IC/BPS bladder [21].

Furthermore, the present study used Western blot analysis to confirm the molecular mechanism of chronic inflammatory signalling and apoptosis. The results showed tryptase and Bax protein expression increased in the recurrent UTI specimens compared with the control specimen. Tryptase is the most abundant secretory granule-derived serine proteinase contained in mast cells that has been used as a marker for mast cell activation [15], [22]. One previous study has shown that p38α mediates apoptosis through phosphorylation of downstream molecules, which could be a common regulatory point for cell death [23]. Bax is one of the pro-apoptotic proteins known to mediate the apoptotic process. Previous studies have indicated that p38 mitogen-activated protein kinase (MAPK) activation has been implicated in inflammation and fibrosis and in mediating apoptosis in different cell types in various species [24], [25]. It is well known that the p38 MAPK pathway participates in the apoptosis process through the regulation of p53 activation. The role of p53 in inducing apoptosis is through the mechanisms involving Bad and Bax, which are the main factors that mediated mitochondrial dysfunction and cell apoptosis [26], [27].

Our previous study has demonstrated a significant correlation between mast cell activation and urothelial cell apoptosis in IC/BPS bladder [5]. Moreover, important research showed that apoptosis of urothelial cells in patients with IC/BPS could result from upregulation of inflammatory signals, including p38 mitogen-activated protein kinase and tumor necrosis factor-α [21]. The findings from the study demonstrated that apoptosis was present in the urothelium of patients with recurrent UTI and is possibly mediated by the inflammatory pathway.

To our knowledge the urothelial homeostasis in recurrent UTI has not been reported before. It is possible that chronic inflammation might reside in the bladder wall, which might also contribute to urothelial dysfunction and defective barrier function, then UTI might be easy to recur in these patients.

### Conclusions

Our preliminary results showed that chronic inflammation, urothelial cell apoptosis and impairment of barrier function of urothelial cells could be the underlying pathophysiology of recurrent UTI in women. Chronic inflammation might reside in the bladder wall after resolution of UTI, which might contribute to urothelial dysfunction and defective barrier function and UTI will be easy to recur in these patients. This evidence provides a new focus for clinical management and future research of recurrent urinary tract infection.
